# Tele-education model for primary care providers to advance diabetes equity: Findings from Project ECHO Diabetes

**DOI:** 10.3389/fendo.2022.1066521

**Published:** 2022-12-16

**Authors:** Ananta Addala, Stephanie L. Filipp, Lauren E. Figg, Claudia Anez-Zabala, Rayhan A. Lal, Matthew J. Gurka, Michael J. Haller, David M. Maahs, Ashby F. Walker, for the Project ECHO Diabetes Research Team, Michael Haller

**Affiliations:** ^1^ Department of Pediatrics, Division of Pediatric Endocrinology, Stanford University, Stanford, CA, United States; ^2^ Department of Pediatrics, University of Florida, Gainesville, FL, United States; ^3^ Department of Medicine, Division of Endocrinology, Stanford University School of Medicine, Stanford, CA, United States; ^4^ Stanford Diabetes Research Center, Stanford University, Stanford, CA, United States; ^5^ Department of Health Services Research, Management and Policy, University of Florida, Gainesville, FL, United States

**Keywords:** equity, primary care provider education, disparities, health, type 1 diabetes, tele-education

## Abstract

**Introduction:**

In the US, many individuals with diabetes do not have consistent access to endocrinologists and therefore rely on primary care providers (PCPs) for their diabetes management. Project ECHO (Extension for Community Healthcare Outcomes) Diabetes, a tele-education model, was developed to empower PCPs to independently manage diabetes, including education on diabetes technology initiation and use, to bridge disparities in diabetes.

**Methods:**

PCPs (n=116) who participated in Project ECHO Diabetes and completed pre- and post-intervention surveys were included in this analysis. The survey was administered in California and Florida to participating PCPs *via* REDCap and paper surveys. This survey aimed to evaluate practice demographics, protocols with adult and pediatric T1D management, challenges, resources, and provider knowledge and confidence in diabetes management. Differences and statistical significance in pre- and post-intervention responses were evaluated *via* McNemar’s tests.

**Results:**

PCPs reported improvement in all domains of diabetes education and management. From baseline, PCPs reported improvement in their confidence to serve as the T1D provider for their community (pre vs post: 43.8% vs 68.8%, p=0.005), manage insulin therapy (pre vs post: 62.8% vs 84.3%, p=0.002), and identify symptoms of diabetes distress (pre vs post: 62.8% vs 84.3%, p=0.002) post-intervention. Compared to pre-intervention, providers reported significant improvement in their confidence in all aspects of diabetes technology including prescribing technology (41.2% vs 68.6%, p=0.001), managing insulin pumps (41.2% vs 68.6%, p=0.001) and hybrid closed loop (10.2% vs 26.5%, p=0.033), and interpreting sensor data (41.2% vs 68.6%, p=0.001) post-intervention.

**Discussion:**

PCPs who participated in Project ECHO Diabetes reported increased confidence in diabetes management, with notable improvement in their ability to prescribe, manage, and troubleshoot diabetes technology. These data support the use of tele-education of PCPs to increase confidence in diabetes technology management as a feasible strategy to advance equity in diabetes management and outcomes.

## Introduction

Disparities in diabetes management and outcomes are increasingly recognized as important to address (1–3). The drivers of disparities are multi-layered with contributions from social determinants of health, structural inequities, insurance policies, and health care providers (1, 4). Many individuals with diabetes do not consistently receive subspecialty care from an endocrinologist or diabetologist and rely on primary care providers (PCPs) for their diabetes management (5, 6). However, PCPs are not trained on the specifics of diabetes management, including the nuances of insulin dosing and diabetes technology management. Although PCPs report regularly filling insulin prescriptions for individuals with diabetes, they report low confidence in providing diabetes care, including diabetes technology management (6). Lack of provider confidence in diabetes technology management and provider bias are barriers to technology access and utilization for individuals with diabetes (7–9). Disparities in diabetes management are pronounced in underrepresented groups including individuals who do not receive care from an endocrinologists/diabetologist, those from racial/ethnic minority groups, with public insurance, from low socioeconomic groups, and/or live in rural communities (5, 10–12).

During the COVID-19 pandemic, increase in telehealth utilization, where the endocrinology subspecialty provider engages in direct patient care and management, has become a more commonplace and well-accepted (13–15). While this model has been considered useful in increasing the access to subspecialty care, barriers to telehealth utilization have included limitations due to English proficiency, health literacy, technology access, and broadband/connectivity issues (16–18). Additionally, there’s a well-established shortage of endocrinology subspecialty providers that make it challenging for individuals living with diabetes to reliably and consistently seek medical care from an endocrinologist (6, 19–21). In addition to the shortage of pediatric and adult endocrinology providers, the incidence and prevalence of pediatric and adult diabetes is increasing which further exacerbates the access gap, particularly for underrepresented populations (22–24). The COVID-19 pandemic accelerated telehealth implementation for nearly all diagnoses such that telemedicine is now considered a standard modality for health care delivery (13, 14, 25, 26). That said, telehealth alone is not sufficient to ensure access to standard of care for many individuals living with diabetes. This is especially true when it comes to the democratization of adjuvant pharmacotherapy and access to diabetes technologies.

PCP education is a reliable method to increase comfort with medical management and has been utilized to expand access and reliably deliver standard of care (25–32). Additionally, self-reported improvements in provider confidence are associated with changes in clinical practice and behavior in the medical field at large (33–35). To increase PCP comfort with diabetes management, Project ECHO (Extension for Community Healthcare Outcomes) Diabetes was developed as a tele-education model to democratize diabetes knowledge to PCPs (5, 6, 36–38).The education provided in Project ECHO Diabetes aimed to address general diabetes knowledge, insulin and diabetes pharmacotherapy, diabetes technology management, and psychosocial considerations. In this analysis, we aim to evaluate the impact of Project ECHO Diabetes on PCP confidence in management of diabetes. We hypothesized that with systematic education, PCPs will have an increased confidence in all domains of diabetes management.

## Materials and methods

### Project ECHO Diabetes

University of Florida and Stanford University (research “Hubs”) expanded the existing Project ECHO^®^ model (25–28) in collaboration with the University of New Mexico to develop Project ECHO Diabetes, a tele-education program that aimed to train health care providers in underserved areas of California and Florida with a specific focus on recruiting PCPs from federally qualifying health centers and community health centers. The Project ECHO Diabetes program provided participating health centers (“spokes”; Appendix B lists all participating research spokes) and their providers tele-education with continuing medical education credits for participation that details diabetes management. The Project ECHO Diabetes program implemented a stepped-wedge study design with staggered control and intervention periods for new participating spokes. Provider outcomes were collected at baseline, six and 12 months, marking the study end.

### Project ECHO Diabetes curriculum

The Project ECHO Diabetes Curriculum was curated and developed by the multi-disciplinary Hub teams at University of Florida and Stanford University, comprised of pediatric and adult endocrinologists, registered nurses, Certified Diabetes Care and Education Specialists, pharmacists, diabetes psychologists, exercise physiologists, and social workers, to name a few. Education first begins during an orientation session which included providing continuous glucose monitoring (CGM) to providers to experience wearing a CGM and an overview of key diabetes management areas. After the onboarding process, weekly tele-education sessions included a 20 to 30 minute didactic session by experts from the Hub team or affiliated experts to deliver content on diabetes management consistent with the American Diabetes Association Standards of Care (39–44). **Table 1** outlines 24 weekly sessions carried out over the 6-month curriculum. Didactic sessions are followed by a case presentation from the spokes, where the presenting provider details a clinical conundrum in the management of diabetes. Feedback is initially solicited from the spokes, followed by input from the Hub. These tele-education clinic sessions are accredited by both universities for Continuing Medical Education credits, which are provided at no cost to attendees.

**Table 1:** Project ECHO Diabetes Curriculum.

The Pillars of Success: Knowledge, Community, & Resilience	Initiating Insulin Pump Therapy
Continuous Glucose Monitoring Systems	Introducing Diabetes Technology to Patients
Glycemic Targets and Glucose Monitoring	Screening for Depression and Diabetes Burnout
T2D Management in Established Atherosclerotic Cardiovascular Disease	Carb Counting and Dietary Management in T1D
T2D Management in Heart Failure and Chronic Kidney Disease	Making a Diagnosis of Diabetes in Primary Care
T2D Management: Promoting Weight Loss Using Glucose-Lowering Medications	Diabetes and Hypertension Management
T2D Management: Strategies to Minimize Hypoglycemia	Dyslipidemia and Diabetes
Using and Interpreting Data from CGMs	Exercise Strategies in T1D and T2D
Initiating Insulin and Dose Calculations in T1D and T2D	Motivational Interviewing
T2D Management: When Medication Cost is a Major Issue	Diabulimia and Disordered Eating
Types of Insulin Analogs	Diabetes and Complications Screenings
Calculating Insulin Doses for T1D and T2D: Case Based Approach	Sick Day Management and Severe Hyperglycemia

Six-month curriculum of 24 sessions occurring every week.

### Survey methodology

Participating providers were invited to take pre- and post-intervention surveys administered *via* REDCap^©^ (Research Electronic Data Capture) prior to and after the 6-month intervention. REDCap is a secure online-enabled platform which allowed surveys to be sent directly *via* email and taken electronically using any web browser.

The survey content was designed to assess knowledge (using 18 multiple-choice test questions) and confidence (using 28 questions with a 4-point Likert scale response set ranging from ‘not at all confident’ to ‘extremely confident’) in diabetes care. Domains of confidence included: general diabetes management, insulin management, diabetes technology, and psychosocial management. Providers were offered surveys at all three data collection windows. Due to variable kick-off dates for the implementation of Project ECHO Diabetes for cohorts 1 and 2, providers were offered either two pre-surveys and one post survey or one pre-survey followed by two post surveys at 6-month intervals.

### Analysis

The *a priori* primary outcome of this analysis was changes in provider confidence and the secondary outcome was improvement in provider knowledge. To account for variable survey administration, data were extracted on an individual-basis from the earliest pre-test and the latest post-test. Only those providers with at least one pre- and one post-survey were included in the statistical analysis to evaluate change in provider confidence, and acquisition of knowledge with the Project ECHO Diabetes curriculum.

All data management and analyses were conducted using SAS 9.4^®^ (Cary, NC). Descriptive statistics are reported for provider and practice characteristics. Paired pre and post-intervention comparisons were made among new spoke participants to evaluate for change in confidence and knowledge using McNemar’s test; a predetermined alpha of 0.05 was used to evaluate statistical significance. Aggregate data are presented descriptively to elucidate the scope of adult referrals to endocrinology and CGM/insulin pump prescriptions pre- and post-intervention among all responding providers.

Project ECHO Diabetes received institutional review board (IRB) approval by University of Florida and Stanford University.

## Results

In total, 274 providers (123 from California, 145 from Florida) were referred to Project ECHO Diabetes for participation by their Spoke site’s ECHO Champion and 116 providers completed at least one survey (**Table 2**). Providers were predominately PCPs (63.7% physicians and 29.1% advanced practice providers). Responding providers have been in practice for a median of 7.5 years (IQR 2.5,16.5). Across the three data collection time windows, 110 providers completed a pre-intervention survey, 58 providers completed a post-intervention survey, and among those, 52 providers completed both a pre- and post-intervention survey.

**Table 2:** Provider characteristics.

Total providers responding to either survey	116
Credentials:	
Physician (MD/DO)	68 (63.7)
Physician Assistant	4(3.6)
Advanced Practice Providers	32 (29.1)
Registered Nurse	2 (1.8)
Other	2 (1.8)
Years in Practice, years*	7.5 [2.5, 16.5]
# Adult patients with diabetes treated annually**	150.0 [58.0, 426.0]
# Adult patients with insulin-requiring diabetes treated annually***	50.0 [20.0, 100.0]
**N Completed Pre-Survey**	110
**N Completed Post-Survey**	58
**N Completed both Pre- and Post-Survey**	52

* n=108, presenting median years [IQR].

** n=98, presenting median number [IQR].

*** n=97, presenting median number [IQR].

When evaluating data for those providers who had a pre- and post-intervention survey available (n=52), providers demonstrated an improvement in confidence to manage diabetes in all the surveyed questions with 64.3% of improvements reaching statistical significance (**Table 3**). Despite functioning as a primary diabetes provider, 43.8% of surveyed providers felt confidence in being a diabetes resource for the community as well as for other providers and clinics. However, after Project ECHO Diabetes, this confidence increased to 68.8% (p=0.005). Provider confidence increased in general diabetes management including collecting diabetes-focused health history (pre vs post: 78.4% vs. 94.1%, p=0.01) and incorporating diabetes guidelines into clinical care (pre vs post: 72.6% vs. 86.3%, p=0.02).

**Table 3:** Providers report confidence on a variety of diabetes-related items.

Reporting moderately or extremely confident	N†	Pre-survey	Post-survey	p-value
General Diabetes Management				
**Serve as an insulin-requiring diabetes resource for other providers and clinics in my community**	48	21 (43.8)	33 (68.8)	**0.0047**
**Collect a diabetes-focused health history for patients with insulin-requiring diabetes**	51	40 (78.4)	48 (94.1)	**0.0114**
**Incorporating most current American Diabetes Association (ADA) Standard in Medical Care of Diabetes guidelines into your practice**	51	37 (72.6)	44 (86.3)	**0.0196**
Review blood glucose data of patients	51	43 (84.3)	47 (92.2)	0.1025
**Discuss complications related to insulin-requiring diabetes and how to avoid them**	51	36 (70.6)	47 (92.2)	**0.0023**
**Counsel patients about effects of alcohol on insulin-requiring diabetes**	51	33 (64.7)	44 (86.3)	**0.0045**
Counsel patients about effects of exercise on insulin-requiring diabetes.	49	37 (75.5)	42 (85.7)	0.1655
Demonstrate empathy towards patients with insulin-requiring diabetes	49	46 (93.9)	48 (98.0)	0.1573
**Educate clinic staff about patients with insulin-requiring diabetes**	48	30 (62.5)	41 (85.4)	**0.0023**
**Prescribe oral adjunct therapy (SGLT2 inhibitors and GLP-1 agonists)**	51	31 (60.8)	40 (78.4)	**0.0067**
**Identify contraindications to diabetes medications**	50	26 (52.0)	44 (88.0)	**<0.0001**
Insulin Management				
**Manage patients with insulin-requiring diabetes in your primary care setting**	51	32 (62.8)	43 (84.3)	**0.0023**
**Manage basic insulin therapy in patients with insulin-requiring diabetes**	51	35 (68.6)	44 (86.3)	**0.0067**
**Manage basal/bolus insulin therapy in patients with insulin-requiring diabetes**	50	29 (58.0)	40 (80.0)	**0.0023**
Diabetes Technology				
**Determine which patients with insulin-requiring diabetes would benefit from continuous glucose monitor (CGM) device.)**	50	26 (52.0)	39 (78.0)	**0.0016**
**Utilize and interpret continuous glucose monitoring (CGM) data and provide recommendations to my patients with insulin-requiring diabetes**	51	20 (39.2)	34 (66.7)	**0.0017**
**Prescribe a continuous glucose monitor (CGM)**	51	21 (41.2)	35 (68.6)	**0.0010**
Determine which patients with insulin-requiring diabetes would benefit from insulin pump therapy	51	15 (29.4)	22 (43.1)	0.0896
**Manage patients with insulin-requiring diabetes on insulin pump therapy**	48	10 (20.8)	18 (37.5)	**0.0455**
**Manage patients with insulin-requiring diabetes on insulin pump hybrid-closed loop therapy (i.e., Medtronic 670G System, Tandem Control-IQ)**	49	5 (10.2)	13 (26.5)	**0.0325**
Psychosocial Management				
**Assess a patient’s diabetes health literacy (i.e., counting carbohydrates and calculating insulin doses)**	50	25 (50.0)	40 (80.0)	**0.0006**
Provide social support resources to my patients with insulin-requiring diabetes	49	28 (57.1)	34 (69.4)	0.1336
Identify social barriers for my patients with insulin-requiring diabetes. (Social barriers could include financial obstacles like lack of transportation for clinic visits or social support obstacles like lack of family or friendship networks)	50	35 (70.0)	42 (84.0)	0.0707
Provide appropriate interventions for overcoming social barriers for my patients with insulin-requiring diabetes	49	25 (51.0)	33 (67.4)	0.0736
**Identify symptoms of diabetes distress in my patients with insulin-requiring diabetes.**	49	28 (57.1)	41 (83.7)	**0.0046**
Identify depression using the PHQ-8/9 scale and recommend evidence based depression treatment	49	43 (87.8)	47 (95.9)	0.1025
Help make diabetes supplies more affordable and accessible to my patients with insulin-requiring diabetes	50	24 (48.0)	32 (64.0)	0.0881
Help make continuous glucose monitor (CGM) devices affordable for my patients with insulin-requiring diabetes or to be covered by my patients’ health insurance coverage	49	13 (26.5)	20 (40.8)	0.1083

†N Reported represents participants responding to a particular question in both the pre and post surveys.

Bold indicates a statistically significant p-value at the predetermined level α=0.05 for New Spoke Provider Pre-Post Moderate/Extreme Confidence.

McNemars Testing to used to evaluate pre-post paired data.

Providers with Pre-Post data with 4-point Likert scale: (1) Not at all Confident (2) Somewhat Confident (3) Moderately Confident (4) Extremely Confident.

Providers also felt more comfortable with insulin management in the primary care setting (pre vs post: 62.8% vs. 84.3%, p=0.002) as well as in basic insulin management (pre vs post: 68.6% vs. 86.3%, p=0.007) and management of basal/bolus therapy (pre vs post: 58.0% vs. 80.0%, p=0.002). Providers had significant improvement in their comfort with oral adjuvant therapy in the treatment of insulin-requiring diabetes including the use of GLP1 agonists and SGLT2 inhibitors (pre vs post: 60.8% vs. 78.4%, p=0.007) and endorsed an ability to identify contraindications in diabetes medications (pre vs post: 52.0% vs. 88.0%, p<0.0001).

When compared to pre-intervention, provider confidence increased in nearly all aspects of CGM management post-intervention, including determining which patients would benefit from CGM (52.0% vs. 78.0%, p=0.002), prescribing CGM (41.2% vs. 68.6%, p=0.001), and interpreting CGM (39.2% vs. 66.7%, p=0.002). Similarly, providers endorsed increased confidence with managing insulin pumps (28.8% vs. 37.5, p=0.046) including hybrid closed loop systems (10.2% vs. 26.5%, p=0.03).

In addition to improvements in confidence, providers demonstrated an improvement in knowledge pertaining to diabetes management (**Supplemental Table 1**). In particular, provider knowledge increased with respect to calculating insulin doses (pre vs post: 19.2% vs. 48.1%, p=0.002), understanding the impact of metformin in insulin-requiring diabetes (pre vs post: 57.7% vs. 75.0%, p=0.04), pharmacotherapy for weight management in type 2 diabetes (pre vs post: 88.5% vs. 96.2%, p=0.046), and identifying function of various CGM reports (pre vs post: 17.3% vs. 46.2%, p=0.046).

In addition to evaluation of pre-post data among those responding, we descriptively assessed prescribing and referral practices for all providers who responded to any survey, including those who didn’t complete both pre and post. **Supplemental Table 2** presents provider confidence for all available pre- and post-intervention surveys (110 and 58 surveys, respectively). Descriptive improvements were observed in all four domains of provider confidence with the greatest improvement gains in CGM prescription (pre: 39.5% and post: 66.7%) and utilization (pre: 38.5% and post: 66.7%). Among providers who completed pre-intervention surveys, 39.3% (n=107 responding) of providers were moderately or extremely comfortable serving as the diabetes resource and post-intervention, 60.7% (n=56 responding) of providers were moderately or extremely comfortable.

We descriptively evaluated prescribing and referral practices for all providers who responded to any survey, including those who didn’t complete both pre and post (**Supplemental Table 3**). Before Project ECHO Diabetes, 15.4% (n=65 responding) PCPs indicated they always (vs. sometimes or never) referred diabetes care to an endocrinologist but after the intervention, only 5.0% (n=20 responding) would always refer care. Pre-intervention, among all responding providers, only 31.3% reported always prescribing CGM, and 7.3% reported always prescribing insulin pumps. Post-intervention, prescription rose to 51.0% for providers always prescribing CGM and 12.8% for always prescribing insulin pumps.

## Discussion

We report success in increasing PCP confidence and knowledge in the management of diabetes in the primary care setting with a tele-education model as a means to improve access to diabetes standard of care. Project ECHO Diabetes was designed with the pillars of modern medical educational framework to include didactics, case presentations, and supervision from experienced diabetes clinicians (25–28). The systematic delivery of tele-education curriculum to PCPs to execute subspecialized medical care delivery has been successful with the Project ECHO^®^ model (25–32), however this model is under-studied with respect to diabetes field with only two active Project ECHO protocols to address diabetes care (5, 30).

By arming PCPs to effectively manage diabetes, the potential exists to bridge disparities in diabetes management and outcomes. PCPs had a significant increase in their confidence to manage insulin for individuals with diabetes, which was a commonly stated barrier before Project ECHO Diabetes was initiated in the clinics. They also better understood the role that metformin plays on insulin dosing and management. This increased confidence in the management of insulin, including comfort with a basal/bolus regimen, is foundational to the intensive insulin therapy which is associated with improvements in short- and long-term diabetes health outcomes. Providers also had an increase in comfort with new and effective adjuvant therapy for type 2 diabetes including SGLT2 inhibitors and GLP-1 agonist which are associated with a decrease in micro- and macro-vascular complications. PCPs increased comfort with the utilization of adjuvant therapy for type 2 diabetes management is likely to improve utilization of these effective pharmacotherapy. An important next step for this research will be to better understand the impact of this increase in provider confidence on improvements in provider knowledge and changes in prescribing practices. Further studies are needed to better understand how PCP knowledge is impacted with increased confidence as well as if and how this translates to improvements in diabetes technology access for their patients.

Utilization of diabetes technology in the management of diabetes is also associated with improvements in glycemic and quality of life outcomes (45). Providers have been identified as a key barrier to diabetes technology uptake and lack of provider knowledge and confidence in prescribing and managing diabetes technology may be a driver (6, 36). The challenges in obtaining and sustaining technology coverage are barriers for providers due to changing coverage policies, cumbersome paperwork, and the time required to complete the paperwork to secure diabetes technology (46, 47). Additional challenges include sustaining technology coverage as well as interpreting 288 daily glucose points and trends. For primary care clinics that do not have specialized staff to support paperwork and prior authorizations, prescribing diabetes technology devices can be time consuming and compound the limited time PCPs have for care delivery. To address provider acceptance of diabetes technology, we facilitated the use of CGM during the Project ECHO Diabetes orientation for all interested providers and discussed the role of provider implicit bias and payer policies in technology utilization. In the Project ECHO Diabetes curriculum, multiple didactic sessions formally covered management of diabetes technology. During the case presentations, diabetes technology was included in recommendations when clinically indicated. In addition to interpreting CGM data and discussing associated insulin dose changes, we also discussed the burden of alarms, skin irritation, and the psychosocial considerations of starting and maintaining diabetes technology use (36, 48, 49). The orientation and curriculum intentionally focused on strategies to optimize coverage of diabetes technology including requirements to secure technology coverage for individuals with public insurance, navigating prior authorizations, modifying EMR templates to include authorization information, and other strategies suggested by spokes to increase the likelihood of payers covering technology. These solutions likely played a role in increasing PCP comfort with diabetes technology.

Provider confidence in the domain of psychosocial management did not change as dramatically as the other domains. The two areas of increased confidence for providers were in their ability to apply the diabetes distress scale and address health literacy in the management of diabetes. The remaining questions in this domain included concepts more commonly covered in primary care such as identifying and addressing social barriers and utilization of evidence-based depression screening as they are part of well adult care (50). In the remaining domains, other questions where there was not a statistical improvement in confidence were areas where providers had started Project ECHO Diabetes with high confidence (for example, demonstrating empathy towards patients living with insulin requiring diabetes). Providers had low confidence in identifying which patients with insulin-requiring diabetes would benefit from insulin pump therapy, and this did not improve with Project ECHO Diabetes. Many providers care predominantly for individuals with type 2 diabetes and there are currently no standard of care or guidelines detailing when an individual with type 2 diabetes may benefit from insulin pump therapy.

These data should be interpreted in the setting of several limitations including selection bias given the response rates and the likelihood that a provider willing to participate in additional education to learn about diabetes care and management may not be representative of all primary care providers. Providers in the study were also confined to practicing in the state of California and Florida, the location of the Hubs, and therefore these data may not be generalizable to other provider practice areas. The assessment of provider confidence and provider knowledge are reported subjectively and not *via* previously validated scales. Additionally, provider knowledge assessment did not extensively evaluate all aspects of diabetes management including insulin pump management. While the scales used in this study were iteratively developed and refined from the Project ECHO Diabetes pilot study, PCP self-efficacy scores may not necessarily correlate with other objective measures of PCP competency and may not reflect changes in diabetes technology prescribing habits. A possible confounding factor is that providers may have received information about diabetes in a setting outside of Project ECHO Diabetes that resulted in improved confidence and knowledge in diabetes care. Despite these limitations, these data offer a promising strategy to increase PCPs confidence to deliver diabetes standard of care.

Training PCPs to deliver subspecialty diabetes care including management of insulin, diabetes technology, and oral adjuvant therapy *via* a tele-education model is effective in improving provider confidence. We demonstrate that this increase in provider confidence translates to an increase in provider knowledge and changes to prescribing practices. Given the increasing burden of diabetes both nationally and globally, partnering with PCPs, who are already caring for individuals with diabetes, to deliver the standard of care in diabetes is a viable path to addressing disparities in access to diabetes care and management.

## Data availability statement

The raw data support the conclusions of this article will be made available by the authors, without undue reservation upon reasonable request.

## Ethics statement

Project ECHO Diabetes received institutional review board (IRB) approval by University of Florida and Stanford University. The patients/participants provided their written informed consent to participate in this study.

## Members of Project ECHO Diabetes Hub Teams:

**University of Florida Diabetes Institute:**

Michael Haller, MD – Principal Investigator, Pediatric Endocrinologist

Ashby Walker, PhD – Co-Principal Investigator and Project Director, Medical Sociologist

Eleni Sheehan APRN, FNP, CDCES – Clinic Manager

Angelina Bernier, MD – Pediatric Endocrinologist

Sarah Westen, PhD – Clinical Health Psychologist

Hannah Stahmer, RD, CDCES – Registered Dietician

William Troy Donahoo, MD, FTOS – Adult Endocrinologist

Xanadu Roque, BA – Research Coordinator

Gabby Malden, BA – Administrative Assistant

Melanie Hechavarria, MPH – Clinical Research Coordinator

**Stanford University:**

David Maahs, MD, PhD – Principal Investigator, Pediatric Endocrinologist

Rayhan Lal, MD – Pediatric/Adult Endocrinologist, Clinic Co-Director

Ananta Addala, DO, MPH – Pediatric Endocrinologist, Clinic Co-Director

Lauren Figg, MSW – Program Administrator

Katarina Yabut, BS – Research Assistant

Noor Alramahi, BA – Assistant Clinical Research Coordinator

Ana Cortes, BS – Clinical Research Coordinator, ECHO Clinic Coordinator

Dessi Zaharieva, PhD – Exercise Physiologist

Marina Basina, MD – Adult Endocrinologist

Katie Judge, ACNS-BS, CDCES – Diabetes Educator

Lety Wilke, RN, MSN, ACNS-BC, BC-ADM, CDCES – Diabetes Educator

Korey Hood, PhD – Diabetes Psychologist

Jessie Wong, PhD – Diabetes Psychologist

Jason Wang, MD, PhD – General Pediatrics, Policy Outcomes & Prevention

Suruchi Bhatia, MD – Pediatric Endocrinologist

Eugene Lewit, PhD – Health Economist

## Clinics Participating in Project ECHO Diabetes Research

**University of Florida – Florida Sites**

Community Health of South Florida

Miami Beach Community Health Centers

Jessie Trice Community Health System

Evara – Adult Practice

Banyan Health Systems

Orange Blossom Family Health Center

Premier Community HealthCare

Tampa Family Health Centers East

Tampa Family Health Centers West

Borinquen Medical Center

Treasure Coast Community Health

Evara – Pediatric Practice

UF Family Medicine Old Town

UF Family MedicineEastside

Health Care Network SWFLWFL

**Stanford University – California Sites**

Humboldt Independent Practice Association

CommuniCare – Salud Clinic

Solano County Family Health Services

Open Door Community Health Centers

Mendocino Coast Clinics

Shasta Community Health Center

Shasta Cascade Health Centers

Santa Rosa Community Health

Hill Country Community Clinic

CommuniCare – Davis Community Clinic

CommuniCare – Hansen Family Health Center

Valley Diabetes and Obesity

St Agnes Medical Center

United Indian Health Services – Potawot Health Village

Harmony Health Medical Clinic

DAP Health

Health Service Alliance

La Clinica

Tahoe Forest Multispecialty Clinics

## Author contributions

AA, DMM, MJH, and AFW contributed to the concept and design of the study. AA, AFW SLF and MJG structured and SLF and MJG completed the analyses. AA wrote the manuscript with critical contributions from DMM, MJH, RAL, LEF, and AFW. All authors contributed to the article and approved the submitted version.

## Supplementary material

Supplementary Table 1 Provider Knowledge: Number of providers with the correct answers as captured by pre- and post- intervention surveys. Corrected answer highlighted in green. †*N Reported represents participants responding to a particular question in both the pre and post surveys.* Bold indicates a statistically significant p-value at the predetermined level α=0.05 for New Spoke Provider Pre-Post Moderate/Extreme Confidence. McNemars Testing to used to evaluate pre-post paired data.

Supplementary Table 2 Providers Report Confidence on a Variety of Diabetes-Related Items Providers with Pre- or Post-intervention data with 4-point Likert scale: (1) Not at all Confident (2) Somewhat Confident (3) Moderately Confident (4) Extremely Confident. †N Reported represents participants responding to a particular question for either the Pre-Survey or Post-Survey.

Supplementary Table 3 Aggregate pre- & post-intervention survey for providers who completed surveys. * N denotes number of respondents for each question. Response rates for each question varied by which questions providers chose to complete.
