# Supplementary material for: Tele-education model for primary care providers to advance diabetes equity: Findings from Project ECHO Diabetes
Source: Front Endocrinol (Lausanne). 2022 Dec 16;13:1066521. doi: 10.3389/fendo.2022.1066521 (PMC9800890; doi:10.3389/fendo.2022.1066521)
Supplement: Supplementary Table 1 — Provider Knowledge: Number of providers with the correct answers as captured by pre- and post- intervention surveys. Corrected answer highlighted in green. †N Reported represents participants responding to a particular question in both the pre and post surveys. Bold indicates a statistically significant p-value at the predetermined level α=0.05 for New Spoke Provider Pre-Post Moderate/Extreme Confidence. McNemars Testing to used to evaluate pre-post paired data. [file DataSheet_1.docx]

**Supplemental Table 1. Provider Knowledge**: Number of providers with the correct answers as captured by pre- and post- intervention surveys. Corrected answer highlighted in green.

| **Questions** | **N†** | **Pre-Survey** | **Post-Survey** | **p-value** |
| --- | --- | --- | --- | --- |
| **Insulin & Medications** |  |  |  |  |
| The duration of Basaglar insulin is: 20 to 24 hours | 52 | 45 (86.5) | 43 (82.7) | 0.5637 |
| **You establish care of a 22-year old 65kg (140 lb) female with Insulin-requiring diabetes who was diagnosed 10 years ago. You are reviewing her insulin doses. Which of the following insulin regimens is most reasonable based on age and weight: Lantus 25 units q morning; 1 unit : 7 grams carbohydrate coverage** | **52** | **10 (19.2)** | **25 (48.1)** | **0.0018** |
| You are reviewing carbohydrate counting and insulin administration with your 25-year old patient with Insulin-requiring diabetes. With respect to insulin and meal timing, the following advice is reasonable to provide your patient: “Inject your insulin 10 to 30 minutes before your meal depending on your blood sugar value.” | 52 | 31 (59.6) | 28 (53.9) | 0.4386 |
| **With respect to Metformin management in Insulin-requiring diabetes, which of the following statements are correct: Metformin decreases total daily insulin requirements** | **52** | **30 (57.7)** | **39 (75.0)** | **0.0389** |
| A patient with Insulin-requiring diabetes is transitioning from multiple daily injections of insulin to continuous subcutaneous insulin infusion. She has been on 100 units per day. At mealtime, the pump should be programmed to deliver 1 unit of insulin for every ____ grams of carbohydrates: 5 | 52 | 10 (19.2) | 15 (28.9) | 0.2253 |
| If the patient in on a total daily insulin dose of 100 units, then the pump should be programmed to deliver an additional 1 unit of insulin for approximately every ___ mg/dl of glucose above the pre-meal target 18 | 52 | 23 (44.2) | 23 (44.2) | 1.0000 |
| You are intensifying medical management in a patient with type 2 diabetes (T2D) who is currently on basal insulin (1 unit/kg/day), metformin, and sulfonylurea therapy. Basal insulin was added at the last visit 6 months ago. The patient’s most recent A1c level was 9.5% and he is having blood sugars over 200 mg/dL after meals. An appropriate next step would be to: Stop sulfonylurea and add prandial insulin or GLP-1 agonist | 52 | 42 (80.8) | 42 (80.8) | 1.0000 |
| Which of the following classes of type 2 diabetes medications is MOST likely to cause symptoms of hypoglycemia during periods of extra activity (exercise)? Sulfonylureas | 52 | 45 (86.5) | 45 (86.5) | 1.0000 |
| **Your patient is a 42 year-old female with new-onset type 2 diabetes. Her A1c is 8.5% and her BMI is 34. She has hypertension and hyperlipidemia with no other known complications. Which of the following agents would be most helpful for your patient to lose weight? Dulaglutide (Trulicity)** | 52 | 46 (88.5) | 50 (96.2) | **0.0455** |
| **Blood Glucose Monitoring and Continuous Glucose Monitor (CGM)** |  |  |  |  |
| Reasonable blood glucose targets before and 2 hours after a meal for a 16-year-old female with Insulin-requiring diabetes would be: Before meal: 80 -130mg/dl; after meal: less than 180 mg/dl | 52 | 40 (76.9) | 37 (71.2) | 0.4669 |
| **In reviewing and evaluating continuous glucose monitor (CGM) data, the following report is a standardized report that allows you to review and analyze data regardless on the brand of the device: AGP Report** | 52 | 9 (17.3) | 24 (46.2) | **0.0003** |
| 12. You recently placed 4 patients on a continuous glucose monitor (CGM) and are reviewing and comparing the 14-day reports of each patient. Which patient profile is preferable based on average blood sugar and standard deviation? 150 +/-50 | 52 | 36 (69.2) | 30 (57.7) | 0.2207 |
| 13. When considering “Beyond HbA1c goals” and evaluating continuous glucose monitor (CGM) reports what is a reasonable goal for hypoglycemia in non-pregnant individuals with type 1 diabetes? < 4% hypoglycemia | 52 | 35 (67.3) | 35 (67.3) | 1.0000 |
| When considering “Beyond HbA1c goals” and evaluating continuous glucose monitor (CGM) reports what is a reasonable target for time in rage (TIR) goal for pregnant individuals with type 1 diabetes or type 2 diabetes? 63-140mg/dL | 52 | 23 (44.2) | 16 (30.8) | 0.1266 |
| Which is of the following is NOT a clinical indication of medical necessity for instituting continuous glucose monitoring?  Pregnancy. Recurring episodes of severe hypoglycemia.  Dawn phenomenon where fasting blood glucose level often exceeds 200 mg/dl. Day-to-day variations in work schedule, mealtimes and/or activity level, which confound the degree of regimentation required to self-manage glycemia with multiple insulin injections. None of the above. | 52 | 25 (48.1) | 31 (59.6) | 0.2008 |
| **Complications** |  |  |  |  |
| According to the ADA guidelines, for most patients with diabetes, the target blood pressure should be less than: 120/80 | 52 | 16 (30.8) | 15 (28.9) | 0.8273 |
| According to the ADA guidelines, for most patients with diabetes, the target LDL cholesterol level should be less than: 100 | 52 | 30 (57.7) | 30 (57.7) | 1.0000 |
| When should individuals with type 2 diabetes have an **initial** dilated and comprehensive eye examination by an ophthalmologist or optometrist? Shortly after diagnosis with diabetes | 52 | 38 (73.1) | 44 (84.6) | 0.1336 |

† *N Reported represents participants responding to a particular question in both the pre and post surveys*

**BOLD** indicates a statistically significant p-value at the predetermined level α=0.05 for New Spoke Provider Pre-Post Moderate/Extreme Confidence

McNemars Testing to used to evaluate pre-post paired data

**Supplemental Table 2. Providers Report Confidence on a Variety of Diabetes-Related Items** *Providers with Pre- or Post-intervention data with 4-point Likert scale: (1) Not at all Confident (***2***) Somewhat Confident (3) Moderately Confident (4) Extremely Confident*

| **Reporting Moderately or Extremely Confident** | **N† Pre-Survey** | **Pre-Survey** | **N† Post-Survey** | **Post-Survey** |
| --- | --- | --- | --- | --- |
| **General Diabetes Management** | | | | |
| Serve as an insulin-requiring diabetes resource for other providers and clinics in my community | 107 | 42 (39.3) | 56 | 34 (60.7) |
| Collect a diabetes-focused health history for patients with insulin-requiring diabetes | 109 | 87 (79.8) | 57 | 53 (93.0) |
| Incorporating most current American Diabetes Association (ADA) Standard in Medical Care of Diabetes guidelines into your practice | 109 | 72 (66.1) | 57 | 47 (82.5) |
| Review blood glucose data of patients | 109 | 91 (83.5) | 57 | 51 (89.5) |
| Discuss complications related to insulin-requiring diabetes and how to avoid them | 108 | 80 (74.1) | 57 | 51 (89.5) |
| Counsel patients about effects of alcohol on insulin-requiring diabetes | 109 | 73 (67.0) | 57 | 47 (82.5) |
| Counsel patients about effects of exercise on insulin-requiring diabetes. | 108 | 77 (71.3) | 56 | 48 (85.7) |
| Demonstrate empathy towards patients with insulin-requiring diabetes | 108 | 98 (90.7) | 56 | 54 (96.4) |
| Educate clinic staff about patients with insulin-requiring diabetes | 107 | 65 (60.8) | 56 | 45 (80.4) |
| Prescribe oral adjunct therapy, including SGLT2 inhibitors and GLP-1 agonists | 109 | 70 (64.2) | 57 | 43 (75.4) |
| Identify contraindications to diabetes medications | 107 | 60 (56.1) | 57 | 47 (82.5) |
| **Insulin Management** | | | | |
| Manage patients with insulin-requiring diabetes in your primary care setting | 109 | 72 (66.1) | 57 | 47 (82.5) |
| Manage basic insulin therapy in patients with insulin-requiring diabetes | 108 | 79 (73.2) | 57 | 48 (84.2) |
| Manage basal/bolus insulin therapy in patients with insulin-requiring diabetes | 107 | 69 (64.5) | 56 | 44 (78.6) |
| **Diabetes Technology** | | | | |
| Determine which patients with insulin-requiring diabetes would benefit from continuous glucose monitor (CGM) device.) | 108 | 55 (50.9) | 57 | 44 (77.2) |
| Utilize and interpret continuous glucose monitoring (CGM) data and provide recommendations to my patients with insulin-requiring diabetes | 109 | 42 (38.5) | 57 | 38 (66.7) |
| Prescribe a continuous glucose monitor (CGM) | 109 | 43 (39.5) | 57 | 38 (66.7) |
| Determine which patients with insulin-requiring diabetes would benefit from insulin pump therapy | 109 | 32 (29.4) | 57 | 25 (43.9) |
| Manage patients with insulin-requiring diabetes on insulin pump therapy | 108 | 21 (19.4) | 55 | 19 (34.6) |
| Manage patients with insulin-requiring diabetes on insulin pump hybrid-closed loop therapy (i.e., Medtronic 670G System, Tandem Control-IQ) | 108 | 15 (13.9) | 56 | 14 (25.0) |
| **Psychosocial Management** | | | | |
| Assess a patient's diabetes health literacy (i.e., counting carbohydrates and calculating insulin doses) | 109 | 52 (47.7) | 56 | 43 (76.8) |
| Provide social support resources to my patients with insulin-requiring diabetes | 106 | 53 (50.0) | 57 | 39 (68.4) |
| Identify social barriers for my patients with insulin-requiring diabetes. (Social barriers could include financial obstacles like lack of transportation for clinic visits or social support obstacles like lack of family or friendship networks) | 108 | 70 (64.8) | 56 | 46 (82.1) |
| Provide appropriate interventions for overcoming social barriers for my patients with insulin-requiring diabetes | 107 | 49 (45.8) | 55 | 36 (65.5) |
| Identify symptoms of diabetes distress in my patients with insulin-requiring diabetes. | 108 | 58 (53.7) | 56 | 45 (80.4) |
| Identify depression using the PHQ-8/9 scale and recommend evidence-based depression treatment (PCP's such as MD, DO, APRN, CDCES, PA's) | 107 | 92 (86.0) | 56 | 53 (94.6) |
| Help make diabetes supplies more affordable and accessible to my patients with insulin-requiring diabetes | 107 | 44 (41.1) | 56 | 34 (60.7) |
| Help make continuous glucose monitor (CGM) devices affordable for my patients with insulin-requiring diabetes or to be covered by my patients' health insurance coverage | 108 | 27 (25.0) | 56 | 22 (39.3) |

† *N Reported represents participants responding to a particular question for either the Pre-Survey or Post-Survey*

**Supplemental Table 3. Aggregate pre- & post-intervention survey for providers who completed surveys**

|  | **N*** | **Always** | **Sometimes** | **Never** |
| --- | --- | --- | --- | --- |
| ***For your adult (ages ≥ 18) patients with insulin-requiring diabetes, please indicate the protocols you follow:*** |  |  |  |  |
| I refer patients with insulin-requiring diabetes to endocrinologists for all their diabetes care |  |  |  |  |
| Pre-Intervention | 65 | 10 (15.4) | 30 (46.2) | 25 (38.5) |
| Post-Intervention | 20 | 1 (5.0) | 12 (60.0) | 7 (35.0) |
|  |  |  |  |  |
| ***For adult patients, I initiate prescriptions and oversee management for the following diabetes medications and supplies:*** |  |  |  |  |
| Continuous glucose Monitors (CGMs) |  |  |  |  |
| Pre-Intervention | 96 | 30 (31.3) | 42 (43.8) | 24 (25.0) |
| Post-Intervention | 49 | 25 (51.0) | 16 (32.7) | 8 (16.3) |
| Insulin Pumps |  |  |  |  |
| Pre-Intervention | 96 | 7 (7.3) | 13 (13.5) | 76 (79.2) |
| Post-Intervention | 47 | 6 (12.8) | 6 (12.8) | 35 (74.5) |

* N denotes number of respondents for each question. Response rates for each question varied by which questions providers chose to complete.

**Appendix A** Members of Project ECHO Diabetes Hub Teams:

**University of Florida Diabetes Institute:**

Michael Haller, MD – Principal Investigator, Pediatric Endocrinologist

Ashby Walker, PhD – Co-Principal Investigator and Project Director, Medical Sociologist

Eleni Sheehan APRN, FNP, CDCES – Clinic Manager

Angelina Bernier, MD – Pediatric Endocrinologist

Sarah Westen, PhD – Clinical Health Psychologist

Hannah Stahmer, RD, CDCES – Registered Dietician

William Troy Donahoo, MD, FTOS – Adult Endocrinologist

Xanadu Roque, BA – Research Coordinator

Gabby Malden, BA – Administrative Assistant

Melanie Hechavarria, MPH – Clinical Research Coordinator

**Stanford University:**

David Maahs, MD, PhD – Principal Investigator, Pediatric Endocrinologist

Rayhan Lal, MD – Pediatric/Adult Endocrinologist, Clinic Co-Director

Ananta Addala, DO, MPH – Pediatric Endocrinologist, Clinic Co-Director

Lauren Figg, MSW – Program Administrator

Katarina Yabut, BS – Research Assistant

Noor Alramahi, BA – Assistant Clinical Research Coordinator

Ana Cortes, BS – Clinical Research Coordinator, ECHO Clinic Coordinator

Dessi Zaharieva, PhD – Exercise Physiologist

Marina Basina, MD – Adult Endocrinologist

Katie Judge, ACNS-BS, CDCES – Diabetes Educator

Lety Wilke, RN, MSN, ACNS-BC, BC-ADM, CDCES – Diabetes Educator

Korey Hood, PhD – Diabetes Psychologist

Jessie Wong, PhD – Diabetes Psychologist

Jason Wang, MD, PhD – General Pediatrics, Policy Outcomes & Prevention

Suruchi Bhatia, MD – Pediatric Endocrinologist

Eugene Lewit, PhD – Health Economist

**Appendix B of Clinics Participating in Project ECHO Diabetes Research**

**University of Florida – Florida Sites**

Community Health of South Florida

Miami Beach Community Health Centers

Jessie Trice Community Health System

Evara – Adult Practice

Banyan Health Systems

Orange Blossom Family Health Center

Premier Community HealthCare

Tampa Family Health Centers East

Tampa Family Health Centers West

Borinquen Medical Center

Treasure Coast Community Health

Evara – Pediatric Practice

UF Family Medicine Old Town

UF Family MedicineEastside

Health Care Network SWFLWFL

**Stanford University – California Sites**

Humboldt Independent Practice Association

CommuniCare – Salud Clinic

Solano County Family Health Services

Open Door Community Health Centers

Mendocino Coast Clinics

Shasta Community Health Center

Shasta Cascade Health Centers

Santa Rosa Community Health

Hill Country Community Clinic

CommuniCare – Davis Community Clinic

CommuniCare – Hansen Family Health Center

Valley Diabetes and Obesity

St Agnes Medical Center

United Indian Health Services – Potawot Health Village

Harmony Health Medical Clinic

DAP Health

Health Service Alliance

La Clinica

Tahoe Forest Multispecialty Clinics
